# Factors associated with dropout at 2 years post-initiation of treatment in the first episode of schizophrenia

**DOI:** 10.4102/sajpsychiatry.v27i0.1657

**Published:** 2021-03-09

**Authors:** Jebril M. Benelmokhtar, Bonginkosi Chiliza, Lebogang Phahladira, Robin Emsley, Laila Asmal

**Affiliations:** 1 Department of Psychiatry, Faculty of Medicine and Health Sciences, Stellenbosch University, Cape Town, South Africa; 2 Department of Psychiatry, Nelson R Mandela School of Medicine, University of KwaZulu-Natal, Durban, South Africa

**Keywords:** adherence, schizophrenia, first episode, dual diagnosis, flupenthixol decanoate, long acting injectable, positive and negative syndrome scale

## Abstract

**Background:**

Prevention of new episodes during the first 2 years after a first episode of schizophrenia (FES) may delay treatment refractoriness and brain morphological changes over time. However, adherence to treatment is characteristically poor in these patients.

**Aim:**

The aim of this study was to examine clinical and sociodemographic factors associated with patient dropout in patients with FES.

**Setting:**

This study was set at inpatient and outpatient services at a psychiatric hospital in the Western Cape, between 2007 and 2011.

**Methods:**

Data were collected as part of a prospective longitudinal study, which followed up patients with FES treated with flupenthixol decanoate. We examined the relationship between treatment adherence and sociodemographic and clinical factors at baseline and at 24 months. Unadjusted and adjusted logistic regression models were used to determine adherence variables.

**Results:**

A total of 62% of patients completed the 24 months of treatment. Participants with FES and a substance use disorder (dual diagnosis) were at greater risk of dropout (*p* = 0.01). On univariate analysis, dual diagnosis participants who dropped out were older (*p* = 0.04) had completed more years of schooling (*p* = 0.001), older age of onset (*p* = 0.02) and higher baseline positive symptoms (*p* = 0.05). On regression analysis, non-completer substance users achieved a higher level of education (odds ratio [OR]: 3.87, confidence interval [CI]: 1.34–11.11, *p* = 0.01).

**Conclusion:**

Substance use disorder was associated with non-adherence to follow up in a cohort of FES patients treated with flupenthixol decanoate. Interventions that take into account age, education and baseline positive symptoms may afford the opportunity to influence adherence and patient outcome.

## Introduction

Schizophrenia is a profoundly disabling and persistent psychiatric disorder that is associated with earlier mortality and considerable disability.^1^ However, schizophrenia is treatable. Decades of research support the use of antipsychotics as the mainstay of treatment for schizophrenia because of its ability to dramatically reduce positive symptoms and prevent relapse.^2^ As proposed in the critical period hypothesis,^3^ therapeutic interventions are most effective if they are administered during critical periods of vulnerability early in the course of the illness.

During these early critical periods of vulnerability, symptomatic and psychosocial deterioration progresses quickly without treatment and thereafter deterioration plateaus.^4^ Therefore, the first 2 years after the first episode of psychosis may have implications for long-term outcomes in terms of symptoms and disability.^5,6^ Prevention of new episodes during this period is vital because it may delay treatment refractoriness, deterioration in symptoms and brain morphological changes over time.^7^ Following the initial challenge of attaining a treatment response in people with schizophrenia experiencing a first episode, a further challenge is to prevent relapse. Continued engagement and adherence to mental health treatment services is therefore vital.

Although poor adherence to treatment is a key predictor of the high relapse rate in first episode psychosis,^8^ adherence to treatment and follow-up is characteristically poor in these patients. The rate of non-adherence in schizophrenia overall is over 50% although studies are largely based in high-income countries.^9^ Adherence studies are lacking in low- to middle-income countries such as South Africa; however, it is likely that non-adherence is higher in these countries because of factors such as limited education, poor mental health literacy and inaccessibility of services.^10^ Results from adherence studies in first episode of schizophrenia (FES) are not much different from that of chronic schizophrenia. Relapse rates at 3 months post-treatment response for a first episode of psychotic illness has been reported at 19% at 3 months^11^ and then rises considerably to 44% and 56% after 6 and 12 months, respectively.^12,13^ A Finnish study of case records of 2588 patients with a first episode of psychosis reviewed over 7 years found that a minority adhere to antipsychotic medication by 2 months post-discharge. Depot medication was associated with a lower risk of hospitalisation compared with oral antipsychotics.^14^

Several factors are associated with poor adherence with follow-up in people with schizophrenia, including patient-related factors, medication-related factors and environmental factors. A history of low educational attainment^15,16^ and never being employed negatively influences adherence rates,^15^ whilst older age is positively associated with adherence.^17^ Prospective and cross-sectional studies have reported associations between substance use disorders (SUDs) and poor adherence in FES.^13,18^ Cannabis use,^19,20^ illicit drug use^13,21^ and alcohol misuse^13,21^ are associated with non-adherence. A patient’s perceived benefit from medication is associated with improved adherence,^22^ whilst a negative attitude towards antipsychotics is a key reason for intentional non-adherence.^23^ A positive relationship with the therapist has been found to be associated with better adherence^22^ and insight impairment is associated with non-adherence to treatment.^24^ Extrapyramidal side effects and other adverse drug reactions such as metabolic syndrome may diminish adherence.^16^ Key environmental barriers to adherence include stigma towards taking medications and lack of family support.^16^

Although a number of studies have identified factors positively and negatively associated with adherence, there are several limitations to the existing literature. Firstly, there is considerable heterogeneity in the methods to measure medication adherence, either objective or patient self-report. Secondly, most studies are cross-sectional surveys or retrospective data analyses with limited characterisation of the sample and a short duration of follow-up. The aim of this study therefore was to examine clinical and sociodemographic factors associated with patient dropout in a clinical study examining patients with FES.

## Methods

### Study design

This study forms part of a larger project, a prospective longitudinal non-comparative study that followed up people with FES who were treated with the lowest effective dose of flupenthixol decanoate medication over 24 months.^25^ This study is a secondary analysis of data collected during the parent study.

### Study setting

The parent study was conducted in Cape Town, South Africa, and FES patients were recruited from inpatient and outpatient services at a psychiatric hospital and surrounding psychiatric clinics in the Western Cape between 2007 and 2011.

### Study sample

As part of the parent study, inclusion criteria for patients were male or female subjects of 16–45 years age, with first episode of psychosis meeting Diagnostic and Statistical Manual of Mental Diseases, Fourth Edition (DSM-IV) diagnostic criteria for schizophrenia,^26^ schizophreniform disorder or schizoaffective disorder and a negative history of current SUD (as confirmed by a urine drug screen and history taking) or a serious medical condition. Exclusion criteria included a lifetime exposure to more than 4 weeks of antipsychotic medication or prior treatment with a long acting injectable (LAI) antipsychotic or intellectual disability (intelligence quotient [IQ] < 70). A total of 126 participants were entered into the parent study.

For the purposes of this study, we focused on dropout of participants. ‘Dropout’ refers to withdrawal from the study that was participant initiated (e.g. did not return for follow-up appointments, self-initiated choice of alternate treatment options). Any withdrawal from the study that was clinician initiated (e.g. non-response, persistent side effects) or based on a collaborative clinician and participant decision (e.g. collaborative choice of alternate treatment) or mortality (one participant died of cancer) was not regarded as a dropout. A total of 16 of the initial 126 participants were excluded from this study because they withdrew from the study in consultation with and on advice of the treating clinician (non-response or side effects) and a further four participants were excluded because one died and three moved to an area with a planned alternate follow-up. Therefore, our final sample composed of 106 participants.

### Data collection

Sociodemographic, anthropometric and clinical data were collected at baseline, obtained from the participant and verified in a separate consultation by the caregiver. The data included age, gender, ethnicity, diagnoses, duration of untreated psychosis and history of SUD. Patient diagnosis of schizophrenia, schizophreniform disorder or schizoaffective disorder and SUD was based on the assessment on intake with the Structured Clinical Interview for DSM-IV (SCID) – Patient Edition.^27^ Although there are some changes to the various levels for SUDs between DSM-IV and DSM 5, the basic definition of SUD remains unchanged.^28^ Symptom severity of schizophrenia was assessed using the Positive and Negative Syndrome Scale (PANSS)^29^ and we used factor analysis-derived symptom domains for positive, negative, depression or anxiety, excitement or hostility and disorganised symptoms.^30^ Depressive symptoms were assessed with the Calgary Depression Rating Scale for Schizophrenia (CDSS).^31^ We administered the Clinical Global Impressions (CGI) Scale as an overall clinician-determined summary measure.^32^ In addition, for overall level of functioning we used Social and Occupational Functioning Assessment Scale (SOFAS)^33^ and the World Health Organization Quality of Life-BREF Scale (WHOQOL-BREF)^34^ for quality of life. Duration of untreated psychosis (DUP) was estimated from the onset of continuous positive psychotic symptoms to initiation of adequate treatment. Adequate treatment was defined as the start of structured treatment with antipsychotic medication. Subjects underwent physical examination. Tolerability measures comprised adverse event (AE) reporting, Extrapyramidal Symptom Rating Scale (ESRS),^35^ weight, height and waist circumference.

Investigators were physicians who were trained in the use of the key assessment instruments and inter-rater reliability testing was conducted periodically (intraclass correlation 0.7 or greater).

### Treatment

All patients were treated with a standard regimen of flupenthixol decanoate intramuscular injection (IMI) medication. There was a washout phase of up to 7 days during which all psychotropic medications were discontinued. Patients were treated with oral flupenthixol 0.5 mg – 4 mg per day for 1 week prior to the first flupenthixol decanoate dose to test for hypersensitivity. The starting dose of flupenthixol decanoate was 10 mg two weekly IMI, with six weekly increments of 10 mg two weekly IMI permitted, to a maximum of 30 mg two weekly IMI. The starting dose could be reduced to 5 mg two weekly IMI in patients younger than 18 years. Other permitted concomitant medications included orphenadrine, trihexyphenidyl or biperiden for parkinsonism or dystonia; propranolol for akathisia; and antidepressants and medication for medical conditions at the investigators’ discretion. Other antipsychotics, mood stabilisers and psychostimulants were not permitted.

### Data analysis

Bivariate associations between categorical exposures and outcome (adherence) were assessed using Pearson’s chi-square tests or Fisher’s exact tests as appropriate. In the case of continuous predictor variables, *t*-tests were used if they are normally distributed and Mann–Whitney tests were used for non-parametric data. We then used unadjusted and adjusted logistic regression to assess for an association between adherence variables that were identified on bivariate analyses. A backward stepwise model was constructed with entry and exit probabilities set at 0.1 and 0.05, respectively. Odds ratios (ORs) and 95% confidence intervals (CIs) were presented in the final model. All analyses were performed using STATA version 15 and statistically significant differences were established at *p* < 0.05.

## Ethical considerations

Approval to conduct the parent study was obtained from the Health Research Ethics Committee of Stellenbosch University (ref: N06/08/148). Study approval was also obtained from management at Stikland Psychiatric Hospital, Tygerberg Hospital and local clinics. Participation in the parent study was voluntary and all participants provided written, informed consent. In the case of minors, written assent and parental consent were obtained.

## Results

### Risk of dropout and factors associated with dropout

Of 106 patients, 40 (38%) dropped out and 66 (62%) completed 24 months of treatment. Reasons for dropout were as follows: two relocated without planning transition to a new service, seven withdrew consent to participate in the study and the remainder were uncontactable or did not give reasons for dropping out. Males made up the majority in both dropout (*n* = 49, 74%) and completion groups (*n* = 30, 75%) (Table 1). Those who dropped out were significantly more likely to have a SUD (*p* = 0.01). There were no other significant differences in demographic and clinical characteristics between those who dropped out and those who completed treatment. Although comorbid SUD was strongly associated with dropout, SUD was also highly prevalent (41%) amongst those participants who completed 24 months of treatment. Therefore, for further analysis, we compared sociodemographic and clinical factors in those participants with dual diagnosis (FES and SUD) who dropped out of treatment versus those who completed treatment.

**TABLE 1:** Demographic and clinical scores for FES study participants who dropped out compared to those who completed 24 months of treatment.

Variables	Dropout (*n* = 40, 38%)				Completers (*n* = 66, 62%)				*t*	*p*
	Mean	SD	*n*	%	Mean	SD	*n*	%		
**Age in years, mean (SD)**	24.65	6.34	-	-	24.91	7.11	0.85	-	-	0.85
Highest grade passed	10.37	1.76	-	-	9.56	2.35	−1.88	-	-	0.06
**Gender, *n*(%)**										
Male	-	-	30	75	-	-	49	74	-	0.93
Female	-	-	10	25	-	-	17	26	-	
**DSM-IV diagnosis *n*( % )**										0.12
Schizophreniform disorder	-	-	17	43	-	-	16	24	-	-
Schizophrenia	-	-	23	57	-	-	49	74	-	-
Schizoaffective disorder	-	-	0	0	-	-	1	2	-	-
Substance abuse**	-	-	16	74			27	41	-	0.01*
Age of illness onset	24.1	6.37	-	-	24.20	7.11	-	-	0.07	0.94
DUP weeks	32.64	37.64	-	-	40.0	50.23	-	-	0.80	0.42
Modal antipsychotic dose	12	4.35	-	-	11.51	3.39	-	-	−0.63	0.52
PANSS total change at 7 weeks	1.15	2.29	-	-	0.93	1.61	-	-	0.36	0.72
**Baseline scores**										
PANSS, total score	90.32	15.06	-	-	94.72	16.07	-	-	1.40	0.16
PANSS, positive factor	17.07	3.41	-	-	17.84	3.03	-	-	1.21	0.23
PANSS, negative factor	18.25	5.27	-	-	19.79	5.47	-	-	1.42	0.16
PANSS depressive factor	9.47	4.16	-	-	9.06	4.32	-	-	−0.48	0.62
PANSS disorganised factors	11.05	3.47	-	-	12.04	2.65	-	-	1.66	0.09
PANSS excite or hostility factor	8.55	3.37	-	-	8.17	3.98	-	-	−0.51	0.61
CDSS total score	3.35	3.96	-	-	3.181	4.1	-	-	−0.21	0.84
CGI (S)	4.82	0.78	-	-	5.07	0.86	-	-	1.49	0.14
SOFAS	42.8	11.97	-	-	45.36	12.13	-	-	1.05	0.29
BIS subscale 1	2.36	1.07	-	-	2.08	1.03	-	-	−1.21	0.23
BIS subscale 2	1.98	0.96	-	-	2.15	1.00	-	-	−0.99	0.32
BIS subscale 3	1.98	0.96	-	-	2.15	1.00	-	-	0.85	0.41
BIS total	6.14	2.25	-	-	5.71	1.93	-	-	−0.54	0.58

DSM-IV, Diagnostic and Statistical Manual of Mental Diseases, Fourth Edition; s.d., standard deviation; PANSS, positive and negative syndrome scale; SOFAS, social and occupational functioning assessment scale; CDSS, Calgary depression scale for schizophrenia; BIS, Birchwood insight scale, CGI, clinical global impression scale; *p*, significance value; *T*-test, for continuous variables; DUP, duration of untreated psychosis.

* , Statistical significance at *p* < 0.05.

** , Data on substance use available for *n* = 89 participants.

### Univariate analysis of factors associated with dropout of treatment in dual diagnosis patients

In our cohort, patients with schizophrenia who also used illicit substances (dual diagnosis) were at greater risk of dropping out. On univariate analyses, those with a dual diagnosis who dropped out differed from those with a dual diagnosis who completed treatment (Table 2). Non-completers with SUD had a later onset of illness (*p* = 0.02), were older at presentation (*p* = 0.04), completed more years of schooling (*p* = 0.001) and had more positive symptoms at baseline (*p* = 0.05) than participant with SUD who completed the study. Participant with SUD who dropped out had poorer insight on the Birchwood Insight Scale (BIS) subscale 2 (awareness of illness) compared with those who completed treatment, but the difference was not significant (*p* = 0.06). Participants with SUD who dropped out where more ill at time of discontinuation compared with those who completed the study as measured by PANSS total score at study exit (*p* < 0.01).

**TABLE 2:** Demographic and clinical scores for dual diagnosis (first episode of schizophrenia and substance use disorders) participants who dropped out compared to those who completed 24 months of treatment.

Variables	Substance users that dropped out (*n* = 17, 39%)				Substance users that completed (*n* = 27, 61%)				*t*	*p*
	Mean	SD	*n*	%	Mean	SD	*n*	%		
**Age in years, mean (SD)**	23.7	5.34	-	-	21.03	2.92	-	-	−2.14	0.04*
Highest grade passed	10.58	1.54	-	-	8.59	2.08	-	-	−3.4	0.001*
**Gender, *n*( % )**									-	0.54
Male	-	-	14	82	-	-	24	89	-	-
Female	-	-	3	18	-	-	3	11	-	-
**DSM-IV diagnosis *n* ( % )**										0.24
Schizophreniform disorder	-	-	8	47	-	-	8	30	-	-
Schizophrenia	-	-	9	53	-	-	19	70	-	-
Schizoaffective disorder	-	-	0	-	-	-	0	-	-	-
Age of illness onset	-	-	20.25	3.52			23.47	5.19	−2.44	0.02*
DUP weeks	42.00	60.12	-	-	21.84	30.33	-	-	1.28	0.21
Modal antipsychotic dose	12.59	3.76	-	-	12.49	3.58	-	-	0.21	0.83
Endpoint: PANSS, total score	45.92	10.08	-	-	56.05	14.29	-	-	−2.75	0.0086
**Baseline scores**										
PANSS, total score	100.44	16.73	-	-	92.17	15.19	-	-	1.65	0.11
PANSS, positive factor	18.29	2.58	-	-	16.41	3.62	-	-	2.01	0.05
PANSS, negative factor	20.70	5.51	-	-	19.7	4.52	-	-	0.62	0.53
PANSS depressive factor	8.22	4.51	-	-	8.35	3.77	-	-	−0.09	0.92
PANSS disorganised factors	13.11	2.48	-	-	12	3.06	-	-	1.31	0.19
PANSS excite or hostility factor	9.85	4.12	-	-	8.70	3.85	-	-	0.91	0.36
CDSS total score	2.47	2.85	-	-	2.47	2.85	-	-	0.17	0.85
CGI (S)	4.94	0.86	-	-	5.33	0.73	-	-	1.64	0.11
SOFAS	38.35	11.0	-	-	39.96	8.69	-	-	0.54	0.59
BIS subscale 1	2.27	0.98	-	-	2.64	1.00	-	-	−1.08	0.28
BIS subscale 2	1.22	1.11	-	-	2	1.24	-	-	−1.94	0.06
BIS subscale 3	1.99	1	-	-	1.90	0.82	-	-	0.29	0.77
BIS total	5.49	1.49	-	-	6.54	1.81	-	-	−1.89	0.07

DSM-IV, Diagnostic and Statistical Manual of Mental Diseases, Fourth Edition; s.d., standard deviation; PANSS, positive and negative syndrome scale; SOFAS, social and occupational functioning assessment scale; CDSS, Calgary depression scale for schizophrenia; BIS, Birchwood insight scale, CGI, clinical global impression scale; *p*, significance value; *T*-test, for continuous variables.

* , Statistical significance at *p* < 0.05.

### Predictors of dropout in dual diagnosis patients

We entered all factors that were associated with dropout in those with SUD at the significance level of *p* < 0.1 into the regression analyses. We found that non-completer participants with SUD had completed more years of schooling (OR = 3.87, 95% CI: 1.34–11.11, *p* = 0.01). No other factor that was significant on univariate analysis remained significant on regression analyses (Table 3).

**TABLE 3:** Predictors of dropout in dual diagnosis first episode of schizophrenia study participants (*n* = 44), *r*^2^ = 0.61.

Variable	OR	CI	*z*	*p*
Number of years of schooling	3.87	1.34 to 11.11	2.51	0.01*
Age in years	2.37	0.27 to 21.26	0.78	0.44
Age of onset	0.78	0.11 to 5.67	−0.24	0.81
PANSS positive score	0.63	0.37 to 1.08	−1.68	0.93
Insight (BIS subscale 2)	4.59	0.91 to 23.20	1.84	0.06

PANSS, positive and negative syndrome scale; BIS, Birchwood insight scale; OR, odds ratio; CI, confidence interval.

* , Statistical significance at *p* < 0.05.

## Discussion

Here, we examined clinical and sociodemographic factors associated with patient dropout in a clinical study of patients with a FES treated over 2 years with flupenthixol decanoate. The most striking finding was that participants with FES and a comorbid SUD were significantly more likely to dropout than those who did not use substances. We did not find an association between any other clinical or demographic variables and dropout. However, we did find that the cohort who used substances and dropped out differed from those who used substances and adhered to follow up.

We did not find an association between non-adherence and sociodemographic factors such as gender, age and the level of education. This is not surprising as previous studies have failed to show a significant relationship between non-adherence and gender,^17,36,37,38^ age^36,38^ and level of education.^17,22,36,37,38^ Furthermore, severity of illness and symptom profile are factors that are inconsistently related to adherence.^18^ In keeping with this, we found that the dropout and completer groups did not differ significantly in terms of symptomatology, insight and level of function.

Substance use disorder was strongly associated with dropout in our cohort (*p* = 0.01) and this finding is comparable to a number of previous studies that report a link between non-adherence and SUD.^13,21,39,40^ In general, Cannabis use^19^ and continuous use of high potency cannabis in particular^20^ are described as risk factors for non-adherence to medication and dropout from treatment. Asher-Svanum et al. found that the second and the third best set of predictors of non-adherence were recent illicit drug use and recent alcohol use, respectively.^21^ A Dublin study found that alcohol misuse at baseline and previous drug misuse predicted non-adherence within 6 months of the first episode of psychosis.^13^ In a cross-sectional study in Ethiopia, social drug use had a statistically significant association with non-adherence.^41^

Although SUD was strongly associated with dropout, SUD was also highly prevalent amongst completers (41%). Why certain dual diagnosis patients dropout whilst others adhere to treatment remains an important unanswered question. Indeed, there are important clinical and resource consequences to non-adherent patients with a dual diagnosis. In a study by Bashir et al., dual diagnosis patients accounted for 57% all hospital readmissions for a schizophrenia cohort and averaged 1.5 readmissions per patient year, potentially contributing to a revolving door phenomenon.^42^

We therefore compared dual diagnosis participants who dropped out with those who remained in treatment and certain key factors emerged. We found on univariate analyses that dual diagnosis patients who dropped out had a later onset of illness, were older at presentation, completed more years of schooling and had more positive symptoms at baseline than those with a dual diagnosis who completed treatment. Other first episode psychosis studies have found that insight and attitude towards medication may predict adherence to people with SUD.^43^ Whilst we did not find that poorer insight was associated with increased dropout in the overall sample, we did find a trend-level association between poor awareness of the illness and dropout in the SUD sample on univariate analyses. This may be an indication that those with SUD and poor insight into their illness may be at particular risk of dropping out of treatment.

The only factor that remained a significant predictor of dropout of treatment in those with SUD, that is, dual diagnosis, on regression analyses was a higher number of years of schooling. The reasons for this are not immediately clear. One suggestion is that participants with higher levels of education with SUD may manage their treatment more independently than those with lower levels of education. Caregivers of participants with a lower level of education may feel that they need to take on a more prominent role in decision-making because of long-standing concern about the patient’s level of functioning.

### Strengths and limitations

The strength of our study is that we prospectively followed up a comparatively large group of carefully characterised FES patients using validated instruments over a 24-month period of treatment. In addition, all participants were assessed by qualified psychiatrists at a research unit and did not have to endure long clinic queues. There were several limitations. Our study was part of a large parent study and dropping out may be reflective of the fact that participants did not want to participate in the study rather than they did not want to take treatment. Studies have reported that coercion in people with schizophrenia may be associated with greater adherence to treatment and participating in a research study potentially adds to the concern regarding coercion. We therefore ensured that the informed consent document was in plain language and was revisited regularly. We also explained to stable patients that should they wish to stop medication before the 24-month period, we would assist them to do so as safely as possible and without prejudice. We also advised that we would assist with the referral process should they wish to obtain treatment elsewhere. The study procedure made it difficult to adjust for the effects of income and system limitations on adherence, two factors that are relevant in South Africa. As part of the ethical requirements of the study, we reimbursed participants for transport costs and medication was provided at no cost. These costs have been cited as factors associated with non-adherence.^44,45^

## Conclusion

In conclusion, these findings highlight the importance of SUD as a risk factor for dropout of treatment. Dual diagnosis patients are vulnerable to non-adherence, but our findings illustrate they are not homogenous in their engagement with follow-up. Our findings provide some indication that a greater number of completed years of education may be a factor associated with dropout in dual diagnosis patients. This, in addition to age of onset of symptoms and severity of positive symptoms, warrants further research in a larger sample. Given that the first episode of illness in schizophrenia is a recognised ‘critical period’,^4^ this period affords an opportunity for interventions that could potentially impact the trajectory of the illness.

## References

[1] World Health Organization. Schizophrenia [homepage on the Internet]. 2020 [cited 2020 Feb 17]. Available from: https://www.who.int/news-room/fact-sheets/detail/schizophrenia

[2] Mueser KT, McGurk SR. Schizophrenia. Lancet. 2004;363:2063–2072. 10.1016/S0140-6736(04)16458-1 15207959

[3] Birchwood M, Todd P, Jackson C. Early intervention in psychosis: The critical-period hypothesis. Int Clin Psychopharmacol. 1998;13:s31. 10.1097/00004850-199804004-00006 9764127

[4] McGlashan TH. Schizophrenia in translation: Is active psychosis neurotoxic? Schizophr Bull. 2006;32(4):609–613. 10.1093/schbul/sbl032 16914639 PMC2632265

[5] Abdel-Baki A, Lesage A, Nicole L, Cossette M, Salvat E, Lalonde P. Schizophrenia, an illness with bad outcome: Myth or reality? Can J Psychiatry. 2011;56(2):92–101. 10.1177/070674371105600204 21333036

[6] Abdel-Baki A, Ouellet-Plamondon C, Malla A. Pharmacotherapy challenges in patients with first-episode psychosis J Affect Disord. 2012;138:S3–S14. 10.1016/j.jad.2012.02.029 22405590

[7] Emsley R, Chiliza B, Asmal L, Mashile M, Fusar-Poli P. Long-acting injectable antipsychotics in early psychosis: A literature review. Early Interv Psychiatry. 2013;7(3):247–254. 10.1111/eip.12027 23342964

[8] Alvarez-Jimenez M, Priede A, Hetrick SE, et al. Risk factors for relapse following treatment for first episode psychosis: A systematic review and meta-analysis of longitudinal studies. Schizophr Res. 2012;139(1–3):116–128. 10.1016/j.schres.2012.05.007 22658527

[9] Lacro JP, Dunn LB, Dolder CR, Leckband SG, Jeste DV. Prevalence of and risk factors for medication nonadherence in patients with schizophrenia: A comprehensive review of recent literature. J Clin Psychiatry. 2002;63(10):892–909. 10.4088/JCP.v63n1007 12416599

[10] Chiliza B. A prospective study of clinical, biological and functional aspects of outcome in first episode psychosis [unpublished dissertation]. Stellenbosch: Stellenbosch University; 2015.

[11] Novak-Grubic V, Tavcar R. Predictors of noncompliance in males with first-episode schizophrenia, schizophreniform and schizoaffective disorder. Eur Psychiatry. 2002;17(3):148–154. 10.1016/S0924-9338(02)00645-4 12052575

[12] Haddad P, Brain C, Scott J. Nonadherence with antipsychotic medication in schizophrenia: Challenges and management strategies. Patient Relat Outcome Meas. 2014;5:43–62. 10.2147/prom.s42735 25061342 PMC4085309

[13] Kamali M, Kelly BD, Clarke M, et al. A prospective evaluation of adherence to medication in first episode schizophrenia. Eur Psychiatry. 2006;21(1):29–33. 10.1016/j.eurpsy.2005.05.015 16460918

[14] Tiihonen J, Haukka J, Taylor M, et al. A nationwide cohort study of oral and depot antipsychotics after first hospitalization for schizophrenia. Am J Psychiatry. 2011;168(6):603–609. 10.1176/appi.ajp.2011.10081224 21362741

[15] Janssen B, Gaebel W, Haerter M, et al. Evaluation of factors influencing medication compliance in inpatient treatment of psychotic disorders. Psychopharmacology. 2006; 187(2): 229–236. 10.1007/s00213-006-0413-4 16710714

[16] Hudson TJ, Owen RR, Thrush CR, et al. A pilot study of barriers to medication adherence in schizophrenia. J Clin Psychiatry. 2004;65(2):211–216. 10.4088/JCP.v65n0211 15003075

[17] Linden M, Godemann F, Gaebel W, et al. A prospective study of factors influencing adherence to a continuous neuroleptic treatment program in schizophrenia patients during 2 years. Schizophr Bull. 2001;27(4):585–596. 10.1093/oxfordjournals.schbul.a006898 11824485

[18] Velligan DI, Sajatovic M, Hatch A, Kramata P, Docherty JP. Why do psychiatric patients stop antipsychotic medication? A systematic review of reasons for nonadherence to medication in patients with serious mental illness. Patient Prefer Adherence. 2017;11:449–468. 10.2147/PPA.S124658 28424542 PMC5344423

[19] Miller R, Ream G, McCormack J, et al. A prospective study of cannabis use as a risk factor for non-adherence and treatment dropout in first-episode schizophrenia. Schizophr Res. 2009;113(2–3):138–144. 10.1016/j.schres.2009.04.018 19481424 PMC2726744

[20] Schoeler T, Petros N, Di Forti M, et al. Effect of continued cannabis use on medication adherence in the first two years following onset of psychosis. Psychiatry Res. 2017;255:36–41. 10.1016/j.psychres.2017.05.009 28521146

[21] Ascher-Svanum H, Zhu B, Faries D, Lacro JP, Dolder CR. A prospective study of risk factors for nonadherence with antipsychotic medication in the treatment of schizophrenia. J Clin Psychiatry;67(7):1114–1123. 2006. 10.4088/JCP.v67n0715 16889456

[22] Löffler W, Kilian R, Toumi M, Angermeyer MC. Schizophrenic patients’ subjective reasons for compliance and noncompliance with neuroleptic treatment. Pharmacopsychiatry. 2003;36(3):105–112 10.1055/s-2003-39985 12806568

[23] Awad AG. Subjective response to neuroleptics in schizophrenia. Schizophr Bull. 1993;19(3):609–618. 10.1093/schbul/19.3.609 7901897

[24] McEvoy JP, Freter SMS, Everett G, et al. Insight and the clinical outcome of schizophrenic patients. J Nerv Ment Dis. 1989;177(1):48–51. 10.1097/00005053-198901000-00008 2535871

[25] Chiliza B, Ojagbemi A, Esan O. et al. Combining depot antipsychotic with an assertive monitoring programme for treating first-episode schizophrenia in a resource-constrained setting. Early Interv Psychiatry. 2016;10(1):54–62. 10.1111/eip.12141 24690088

[26] American Psychiatric Association. Diagnostic and statistical manual of mental disorders: DSM-IV-TR (text revision). Am J Psychiatry: Washington, DC; 2000.

[27] First MB, Spitzer RL, Gibbon M, Williams JBW. Structured clinical interview for DSM-IV-TR axis I disorders, research version, non patient edition. New York, NY: New York State Psychiatric Institute; 2002.

[28] Center for Behavioral Health Statistics and Quality. Impact of the DSM-IV to DSM-5 changes on the national survey on drug use and health. Rockville, MD: Substance Abuse and Mental Health Services Administration; 2016. 30199183

[29] Kay SR, Opler LA, Lindenmayer JP. Reliability and validity of the positive and negative syndrome scale for schizophrenics. Psychiatry Res. 1988;23(1):99–110. 10.1016/0165-1781(88)90038-8 3363019

[30] Emsley R, Rabinowitz J, Torreman M. RIS-INT-35 Early Psychosis Global Working Group. The factor structure for the Positive and Negative Syndrome Scale (PANSS) in recent-onset psychosis. Schizophr Res. 2003;61(1):47–57. 10.1016/s0920-9964(02)00302-x 12648735

[31] Addington D, Addington J, Maticka-Tyndale E. Assessing depression in schizophrenia: The Calgary depression scale. Br J Psychiatry. 1993;163(S22);39–44. 10.1192/S0007125000292581 8110442

[32] Guy W. Clinical Global Impression (CGI). ECDEU Assess. Man Psychopharmacol. Rockville, MD: NIMH Publication; 1976.

[33] American Psychiatric Association. Diagnostic and statistical manual of mental disorders. 4th text revision ed. Washington, DC: American Psychiatric Association; 2000.

[34] Harper A, Power M. Development of the World Health Organization WHOQOL-BREF quality of life assessment. Psychol Med. 1998;28(3):551–558. 10.1017/S0033291798006667 9626712

[35] Chouinard G, Margolese HC. Manual for the Extrapyramidal Symptom Rating Scale (ESRS). Schizophr Res. 2005;76(2–3):247–265. 10.1016/j.schres.2005.02.013 15949657

[36] Acosta FJ, Bosch E, Sarmiento G, et al. Evaluation of noncompliance in schizophrenia patients using electronic monitoring (MEMS®) and its relationship to sociodemographic, clinical and psychopathological variables. Schizophr Res. 2009;107(2–3):213–217. 10.1016/j.schres.2008.09.007 18849150

[37] Aldebot S, Weisman De Mamani AG. Denial and acceptance coping styles and medication adherence in schizophrenia. J Nerv Ment Dis. 2009;197(8):580–584. 10.1097/NMD.0b013e3181b05fbe 19684494 PMC2747048

[38] Jónsdóttir H, Opjordsmoen S, Birkenaes AB, et al. Predictors of medication adherence in patients with schizophrenia and bipolar disorder. Acta Psychiatr Scand. 2012;127(1):23–33. 10.1111/j.1600-0447.2012.01911.x 22900964

[39] Novick D, Maria Haroc J, Suarez D, et al. Predictors and clinical consequences of non-adherence with antipsychotic medication in the outpatient treatment of schizophrenia. Psychiatry Res. 2010;176(2–3):109–113. 10.1016/j.psychres.2009.05.004 20185182

[40] Higashi K, Medic G, Littlewood KJ, et al. Medication adherence in schizophrenia: Factors influencing adherence and consequences of nonadherence, a systematic literature review. Ther Adv Psychopharmacol. 2013;3(4):200–218. 10.1177/2045125312474019 24167693 PMC3805432

[41] Alene M, Wiese MD, Angamo MT, Bajorek BV, Yesuf EA, Tajure Wabe N. Adherence to medication for the treatment of psychosis: Rates and risk factors in an Ethiopian population. BMC Clin Pharmacol. 2012;12:10. 10.1186/1472-6904-12-10 22709356 PMC3416691

[42] Hunt GE, Bergen J, Bashir M. Medication compliance and comorbid substance abuse in schizophrenia: Impact on community survival 4 years after a relapse. Schizophr Res. 2002;54(3):253–264. 10.1016/S0920-9964(01)00261-4 11950550

[43] De Haan L, Van Amelsvoort T, Dingemans P, Linszen D. Risk factors for medication non-adherence in patients with first episode schizophrenia and related disorders; a prospective five year follow-up. Pharmacopsychiatry. 2007;40(6):264–268. 10.1055/s-2007-992141 18030650

[44] Meshach OE, King KM, Fulton JA. Poor adherence to antipsychotics amongst schizophrenia patients in Nigeria. Int J Cult Ment Health. 2014;7(3):246–258. 10.1080/17542863.2013.783091

[45] Teferra S, Hanlon C, Beyero T, Jacobsson L, Shibre T. Perspectives on reasons for non-adherence to medication in persons with schizophrenia in Ethiopia: A qualitative study of patients, caregivers and health workers. BMC Psychiatry. 2013;13:1–9. 10.1186/1471-244X-13-168 23773362 PMC3686587

